# Regulation of Mitochondrial Respiratory Chain Complex Levels, Organization, and Function by Arginyltransferase 1

**DOI:** 10.3389/fcell.2020.603688

**Published:** 2020-12-21

**Authors:** Chunhua Jiang, Balaji T. Moorthy, Devang M. Patel, Akhilesh Kumar, William M. Morgan, Belkis Alfonso, Jingyu Huang, Theodore J. Lampidis, Daniel G. Isom, Antoni Barrientos, Flavia Fontanesi, Fangliang Zhang

**Affiliations:** ^1^Department of Molecular & Cellular Pharmacology, University of Miami Leonard M. Miller School of Medicine, Miami, FL, United States; ^2^Department of Human Genetics, University of Miami Leonard M. Miller School of Medicine, Miami, FL, United States; ^3^Department of Cell Biology, University of Miami Leonard M. Miller School of Medicine, Miami, FL, United States; ^4^Sylvester Comprehensive Cancer Center, University of Miami Leonard M. Miller School of Medicine, Miami, FL, United States; ^5^Institute for Data Science and Computing, University of Miami, Coral Gables, FL, United States; ^6^Department of Neurology, University of Miami Leonard M. Miller School of Medicine, Miami, FL, United States; ^7^Department of Biochemistry & Molecular Biology, University of Miami Leonard M. Miller School of Medicine, Miami, FL, United States

**Keywords:** arginylation, arginyltransferase, mitochondria, biogenesis, respiration, respiratory chain complexes

## Abstract

Arginyltransferase 1 (ATE1) is an evolutionary-conserved eukaryotic protein that localizes to the cytosol and nucleus. It is the only known enzyme in metazoans and fungi that catalyzes posttranslational arginylation. Lack of arginylation has been linked to an array of human disorders, including cancer, by altering the response to stress and the regulation of metabolism and apoptosis. Although mitochondria play relevant roles in these processes in health and disease, a causal relationship between ATE1 activity and mitochondrial biology has yet to be established. Here, we report a phylogenetic analysis that traces the roots of ATE1 to alpha-proteobacteria, the mitochondrion microbial ancestor. We then demonstrate that a small fraction of ATE1 localizes within mitochondria. Furthermore, the absence of ATE1 influences the levels, organization, and function of respiratory chain complexes in mouse cells. Specifically, *ATE1*-KO mouse embryonic fibroblasts have increased levels of respiratory supercomplexes I+III_2_+IV_n_. However, they have decreased mitochondrial respiration owing to severely lowered complex II levels, which leads to accumulation of succinate and downstream metabolic effects. Taken together, our findings establish a novel pathway for mitochondrial function regulation that might explain ATE1-dependent effects in various disease conditions, including cancer and aging, in which metabolic shifts are part of the pathogenic or deleterious underlying mechanism.

## Introduction

Mitochondria are the eukaryotic organelles responsible for oxidative phosphorylation (OXPHOS). They originated from an endosymbiotic event between a respiratory-competent alpha-proteobacteria and an ancient archaea host cell (Bock, 2017). Through evolution, regulatory mechanisms coordinating mitochondrial catabolism have been adjusted to meet the energy and biomass requirements of the eukaryotic cell. At the same time, most of the original bacterial genes have been transferred to the nucleus of the host cell. Currently, the mitochondrial genome (mtDNA) only encodes for a handful of protein-coding genes (8 in yeast, 13 in mammals). The remaining mitochondrial proteome is encoded by the nuclear genome, synthesized in the cytoplasm, and then imported into mitochondria. Therefore, mitochondrial functions are under nuclear control, which involves regulatory processes that are not fully understood.

Arginyltransferase 1 (ATE1) is an evolutionarily conserved enzyme existing in nearly all eukaryotes, with the exception of a few protists (Balzi et al., 1990; Rai and Kashina, 2005; Hu et al., 2006; Rai et al., 2006). Sequence homologs of ATE1 are also found in prokaryotes, although their biological significance is unknown (Graciet et al., 2006). In eukaryotes, ATE1 mediates posttranslational arginylation, the addition of one extra arginine to the target protein. In many cases, arginylation leads to hyper-ubiquitination and rapid degradation of the modified protein, as summarized by the N-end rule theory (Varshavsky, 2011). Mounting evidence suggests that arginylation may act as a response to oxidative stress (Decca et al., 2007; Carpio et al., 2010, 2013; Deka et al., 2016; Kumar et al., 2016). For example, global arginylation activity in cells or tissues is activated by stress caused by reactive oxygen species (ROS), as evidenced by the preferential arginylation of oxidized or misfolded proteins (Ingoglia et al., 2000; Hu et al., 2005). Since mitochondria are a major source of ROS in eukaryotic cells, ATE1 could be an important regulator of mitochondrial redox functions. However, this possibility has yet to be explored.

Several lines of evidence support the idea that the effects of ATE1 activity may be linked to ROS. Cellular phenotypes induced by ATE1 dysregulation often resemble those caused by mitochondrial abnormalities (Saha and Kashina, 2011). Additionally, systematic knockout (KO) of the only gene coding for ATE1 (*ATE1*) leads to embryonic lethality in mouse (Kwon et al., 2002), and postnatal deletion causes rapid weight loss, neurological perturbation, early lethality, and infertility (Brower and Varshavsky, 2009; Kurosaka et al., 2010). Furthermore, tissue-specific knockout (KO) of *ATE1* in mice heart, testis, and central neural system (CNS) leads to cardiomyopathy, infertility, or neural development retardation, respectively (Leu et al., 2009; Kurosaka et al., 2010, 2012; Saha and Kashina, 2011; Wang et al., 2017). Many of these pathological outcomes are consistent with those derived from mitochondrial and metabolic dysregulation and might be explained at the molecular/cellular level by Ate1 activities. For example, *ATE1*-KO leads to multiple metabolic defects, including increased demand for purine supplies and elevated synthesis rate of glycine and alanine, which are often signs of perturbed balance between glycolysis and mitochondrial respiration (Zhang et al., 2015). This hypothesis is further supported by the observation that *ATE1* downregulation is commonly seen in many types of cancer associated with mitochondrial dysfunction (Zhong et al., 2005; Rai et al., 2015).

Recently, we began exploring the genetic interactions between ATE1 and thousands of other genes in the fission yeast model system (*Schizosaccharomyces pombe*) (Wiley et al., 2020). In this study, we observed that ATE1 interacts with only 5% of other yeast genes in a screening library covering 75% of the predicted open reading frames (ORF). Remarkably, more than 10% of those ATE1-interacting genes were related to mitochondria.

This observation motivated us to directly examine the relationship between ATE1 activity, localization, and mitochondrial function. Here, we first analyzed *ATE1* sequences from multiple organisms and determined that eukaryotic *ATE1* may have arisen by gene transfer from alpha-proteobacteria and co-evolved with the function of mitochondria in respiration. Moreover, we show that a small fraction of ATE1 localizes within mitochondria and that ATE1 is required for optimal mitochondrial respiration in both mammalian and budding yeast (*Saccharomyces cerevisiae*) cells. Lastly, we demonstrate that lack of ATE1 in murine cells differentially affects the levels, organization, and function of mitochondrial respiratory complexes. Overall, our finding suggests a hitherto unknown role of ATE1 in the regulation of mitochondrial and cellular energy metabolism.

## Results

### The Alpha-Proteobacterial Origin of ATE1 Links the Protein to Mitochondria

**Figure 1:**
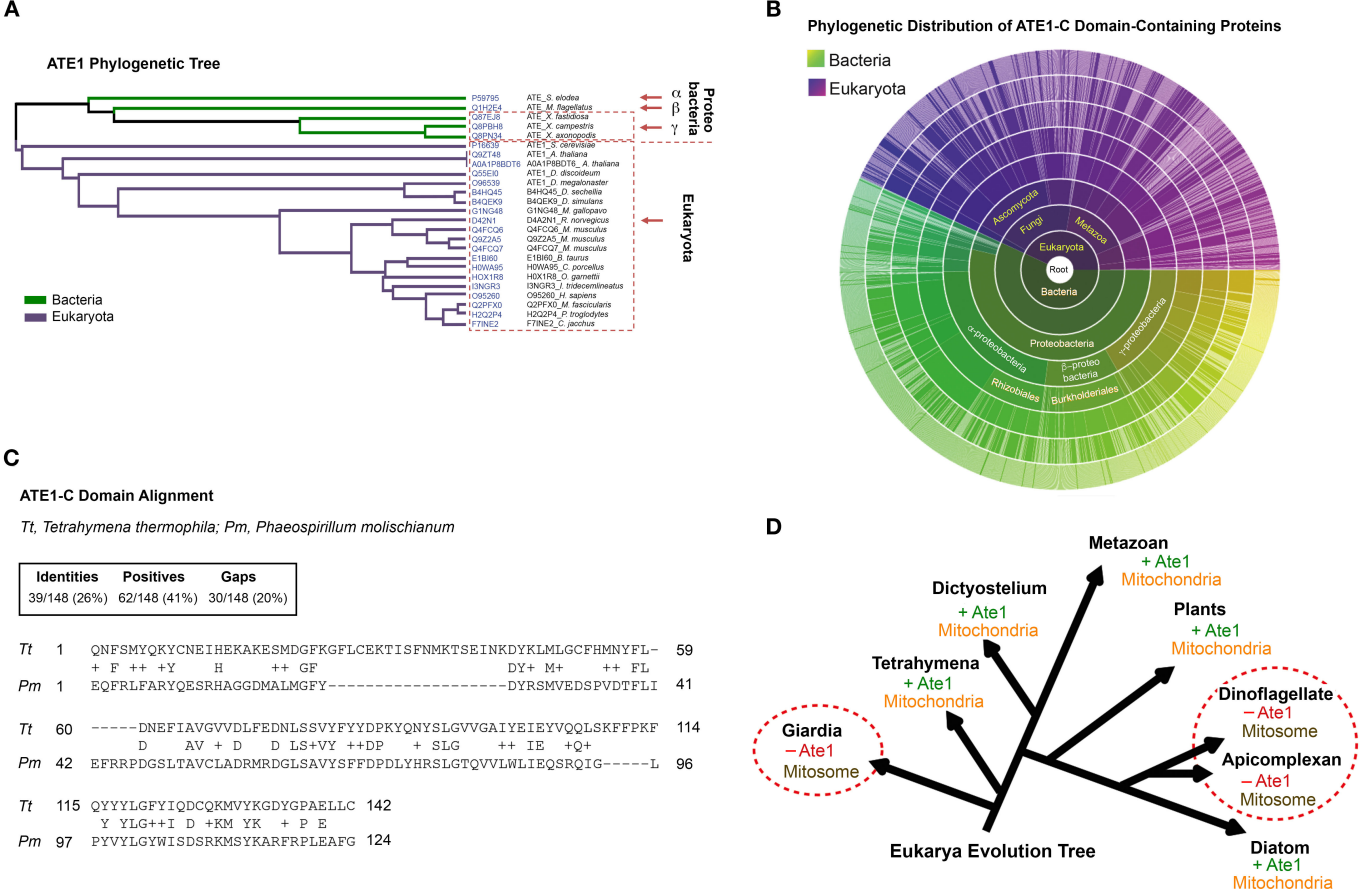
The alpha-proteobacterial origin of ATE1 links the protein to mitochondria. **(A)** Cluster analysis of ATE1 proteins in evolutionary diverse organisms, using the Clustal Omega program on the Uniprot website. The phylogenetic tree is presented as a Notug 2.6 graph. It highlights the clustering of ATE1 from alpha-, beta-, and gamma-proteobacteria, as well as eukaryota. **(B)** Sunburst graph showing the distribution of the catalytic core ATE1-C domain (Pfam ID: PF04377), among 1,796 different species currently known to contain such a sequence encoded in their genomes. The graph was generated with tools from pfam.xfam.org hosted by EMBL-EBI. Yellow-green colors represent different types of bacteria, and purple color represent eukaryotes. No entry from archaea was found in this database. In addition, manual searches for ATE1 homologs on the genome of a representative archaea (*Haloferax volcanii*) using the BlastP function from the NCBI database and the ATE1 amino acid sequence from yeast, mouse, or alpha-proteobacteria as inquiries returned no significant homologous hits. **(C)** Comparison of the amino acid sequences of the core domain of ATE1 (ATE-C domain; Pfam ID: PF04377) between a eukaryote (*Tetrahymena thermophila*, strain SB210) and a alpha-proteobacterium (*Phaeospirillum molischianum* DSM). The sequence alignment was performed with NCBI BLASTp. **(D)** Illustration of the eukarya evolution tree, showing the relationship between the presence of an *ATE1* gene and the mitochondrial development state in several eukaryotic species. The red circles highlight several families (giardia, dinoflagellate, and apicomplexan) in which the absence of ATE1 is accompanied by a loss of respiratory function in mitochondria, organelles that are reduced to a minimized form known as mitosomes.

While a nuclear gene encoded ATE1 in eukaryotes, our search of ATE1 homologs in archaea, where most of the nuclear genome originated (Williams et al., 2013), returned no matches (Figure 1). However, sequence homologs of ATE1 are present in a large population of bacteria, including modern alpha-proteobacteria (Buffet et al., 2020), a close relative to the ancient alpha-proteobacteria that became mitochondria (Figures 1A–C). As such, it is likely that the *ATE1* gene, like many mitochondria-associated genes, was transferred to the nuclear genome during mitochondrial domestication (Janeway and Medzhitov, 2002; Buffet et al., 2020). To gain further insight into the relationship between ATE1 and mitochondria from the perspective of molecular evolution, we examined the status of mitochondrial development and the presence of the *ATE1* gene in several branches of eukaryotes. While almost all eukaryotes contain the *ATE1* gene, two exceptions exist. One is the family of giardia, and the other is the superfamily of dinoflagellates and apicomplexan. Intriguingly, both families lack respiratory-active mitochondria. Instead, they possess mitosomes, a reduced form of mitochondria with minimal functions that cannot perform oxidative phosphorylation (Figure 1D). Since these two families are distally related and separated by many other families that possess ATE1, their loss of ATE1 is unlikely to derive from the same ancestor. For the same reason, their lack of respiratory-competent mitochondria is likely the result of convergent evolution. These data suggest that the presence of ATE1 may be essential for maintaining fully functional mitochondria.

### A Subpopulation of ATE1 Localizes to Mitochondria

**Figure 2:**
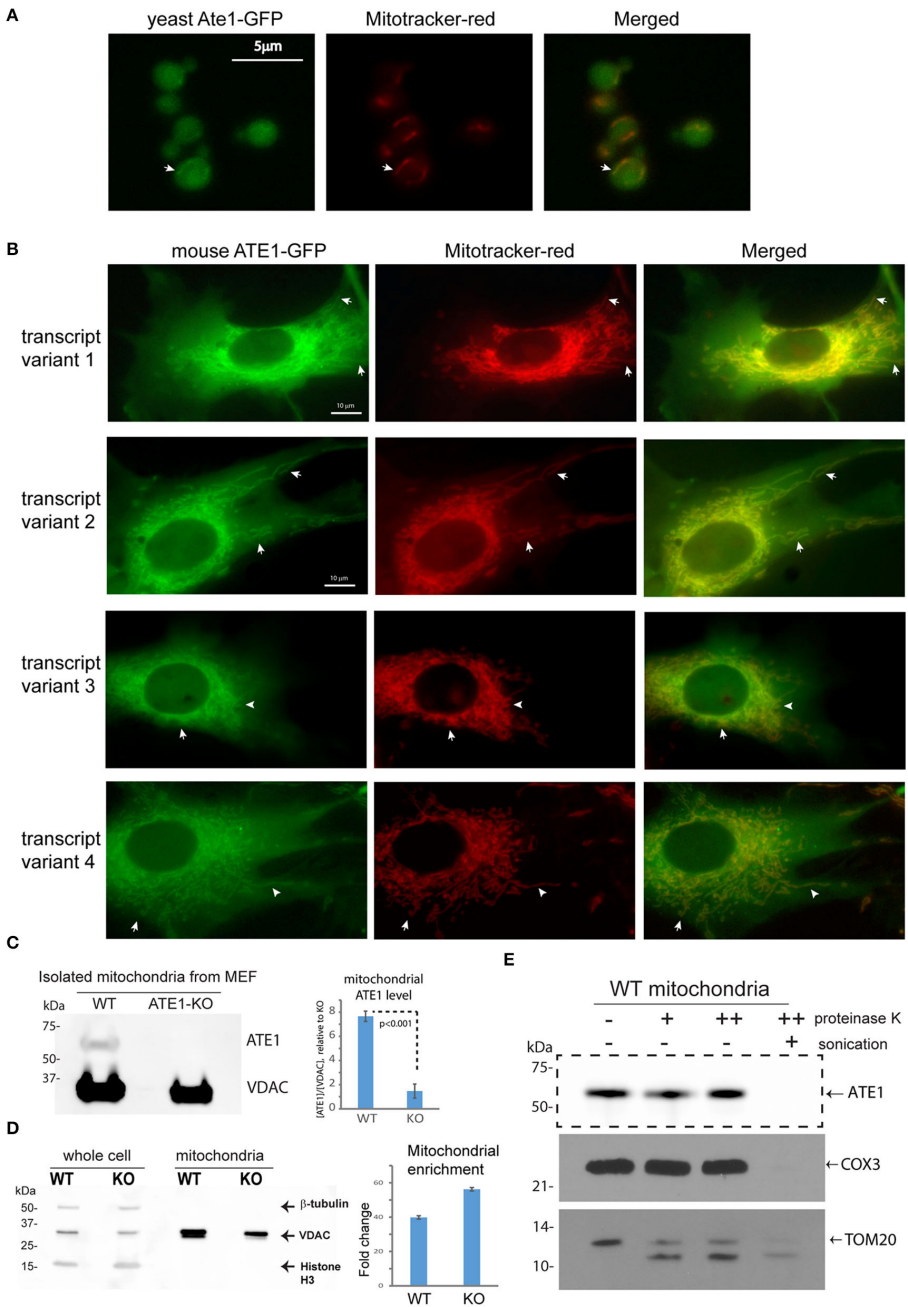
ATE1 locates inside mitochondria. **(A)** The location of yeast Ate1 is traced by a GFP fused to the C-terminus of the endogenous Ate1 on the chromosome of *S. cerevisiae*. The location of Ate1 (shown in green) is then compared to the mitochondria-specific dye, Mitotracker-Red (shown in red). White arrows point to locations where colocalization of Ate1 and mitochondrial structure can be seen. Scale bar indicates 5 micrometers. **(B)** A recombinant mouse ATE1 is fused a C-terminal GFP and stably expressed in mouse embryonic fibroblasts (MEF) with *ATE1-*knockout (referred as “KO” in figure labeling hereon). All four known protein isoforms from splice variants (1, 2, 3, and 4) of the ATE1 gene were examined. The location of GFP-fused ATE1 (shown in green) is compared to mitochondria stained with Mitotracker-Red (shown in red). White arrows point to locations where discrete mitochondrial structures are seen. See also Supplementary Figure 1A for the expression levels of the different ATE1 isoforms. Scale bar indicates 10 micrometers. **(C)** Representative Western blot using a monoclonal antibody that recognizes all four ATE1 isoforms showing a detectable band of endogenous ATE1 in mitochondria from WT MEF, but not from *ATE1-*KO (KO) cells. Antibody for VDAC was used as a loading control on the same blot for mitochondria mass. The experiment was repeated 3 times (*n* = 3). On the right side is the quantification showing the specific signal of ATE1 associated with mitochondria in WT cells. The signal in ATE1-KO cells is used as background control for normalization. The error bar represents standard error of mean (SEM) and the statistical significance was calculated by the *t-test*. See also Supplementary Figure 1B for the estimate of the percentage of mitochondria-associated ATE1 in relation to the total cellular ATE1. **(D)** The purity of mitochondria isolated from WT and *ATE1-*KO cells were examined by Western bot with antibodies against beta-tubulin, VDAC, and histone 3, which are markers for cytosol, mitochondria), and nucleus, respectively. The whole cell lysate was used as control. On the right side shows the chart of quantification of mitochondrial enrichment, which was calculated from three independent repetitions (*n* = 3) by the ratio of VDAC over tubulin in the mitochondrial fraction vs. the whole lysate. The bar graph represents the mean ± SEM. **(E)** Representative Western blot showing the mitochondrial proteinase K protection assay. The signs of “–,” “+,” “++” for proteinase K indicate increasing concentrations (0, 0.32, or 0.64 μg/ml) of proteinase K. An arrow indicates the expected size of ATE1. On the lower side is the Western blot of the digestions of different mitochondria-associated proteins as controls for localizations. These include COX3, which is located inside the mitochondrial matrix, and TOM20, a transmembrane protein anchored on the outer membrane of mitochondria.

Several studies have used fluorescent protein fusions to show the localization of ATE1 in the nucleus or the cytoplasm (Rai and Kashina, 2005; Hu et al., 2006; Rai et al., 2006; Wang et al., 2011). However, the potential localization of ATE1 to mitochondria was not examined directly. To investigate this question, we first utilized a budding yeast (*Saccharomyces cerevisiae*) strain with a genomically-integrated green fluorescent protein (GFP) fused to the C-terminus of the endogenous Ate1 (Ate1-GFP), which showed that a subfraction of the GFP-fused Ate1 colocalizes with Mitotracker red-stained mitochondria (Figure 2A). Next, to test if the mitochondrial localization of ATE1 was organism-specific, we fused a C-terminal GFP to the mouse ATE1, transcript variant 1, a ubiquitously-expressed isoform (Rai and Kashina, 2005; Wang et al., 2011). To avoid the competition of the endogenous protein, this recombinant ATE1 was expressed in *ATE1-*knockout (KO) mouse embryonic fibroblasts (MEF). We detected a fraction of the fluorescent ATE1 colocalizing with the Mitotracker red-stained branched mitochondrial network surrounding the nucleus (Figure 2B). In mice, there are six *ATE1* transcript variants, four of which (transcript variants 1, 2, 3, and 4) are known to be translated into protein isoforms that are >90% identical in sequences (Rai and Kashina, 2005; Hu et al., 2006). We found that the mitochondrial localization of ATE1 is not isoform-specific, because the other three known protein isoforms also appear to localize to mitochondria (Figure 2B; see also Supplementary Figure 1A for the expression level of the tested isoforms).

To further assess the mitochondrial localization of endogenous ATE1, we purified mitochondria from MEF by differential centrifugation and probed for endogenous ATE1 by immunoblotting. We were able to detect a fraction of ATE1 associated with purified mitochondria from wildtype (WT) MEF, while the specificity of the anti-ATE1 antibody was validated by the absence of signals in mitochondria from *ATE1-*KO cells (Figure 2C; see also the purity of the mitochondrial fraction in Figure 2D). Furthermore, a proteinase K protection assay in purified mitochondria showed that the mitochondrion-associated ATE1 is resistant to proteinase K digestion, except when the mitochondrial membranes are disrupted by sonication (Figure 2E). These data indicated that a fraction of ATE1 localizes within mitochondria. To estimate the percentage of ATE1 that is associated with mitochondria, we used a dilution assay of the whole cell and isolated mitochondria to compare the ratios of ATE1 over Voltage-dependent anion channel (VDAC), an established mitochondrial marker. We found that only about 0.5% of total endogenous ATE1 in the cell is associated with mitochondria (Supplemental Figure 1B).

### ATE1 Is Essential for Maintenance of Mitochondrial Morphology and Respiratory Function

**Figure 3:**
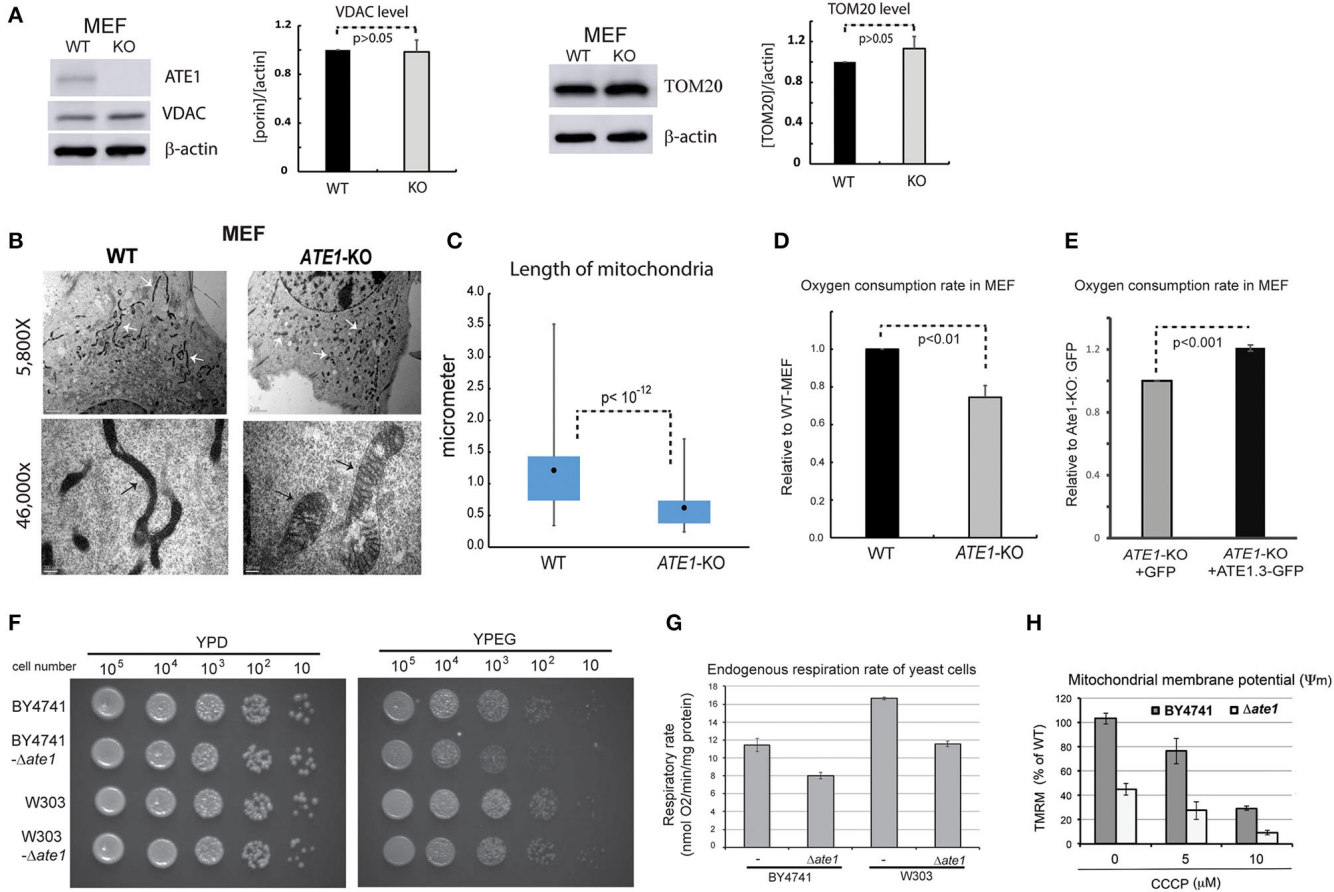
ATE1 is essential for the morphology and function of mitochondria. **(A)** The steady state protein levels of mitochondrial mass markers, VDAC and TOM20, in WT and *ATE1-*KO MEF were examined by Western blot as shown by representative images (left panel) and quantification (right panel) (*n* = 4 for VDAC; *n* = 3 for TOM20). Beta-actin was used as a loading control. **(B)** Morphologies and sizes of mitochondria in WT and *ATE1-*KO MEF shown by negative staining at different magnifications under electron microscopy (5,800x in the upper row and 46,000x in the lower row, as labeled). The scale bars in upper row represent 2 micrometers, while the ones in lower row is 200 nanometers. The white arrows and black arrows indicate a few discrete mitochondria. **(C)** Graph showing box-whisker plots for the lengths of mitochondria in WT and *ATE1-*KO MEF. The quantification of WT cells were based on 115 discrete mitochondrial filaments (*n* = 115) in 6 electron-microscopy (EM) images containing 6 different cells, while for *ATE1-*KO was based on 122 discrete mitochondrial filaments in 4 EM images containing 4 different cells. **(D)** Oxygen consumption rates of the whole cell in WT and *ATE1-*KO MEF. The values in *ATE1-*KO cells were normalized to those in WT cells in each measurement. The bar graph represents the mean ± SEM of four independent measurements (*n* = 4). The *p*-value was calculated by *t*-test. See also Supplementary Figure 2 for representative images of the oxygen consumption curve. **(E)** Similar to **(D)**, except that *ATE1-*KO MEF stably expressing GFP-fused recombinant ATE1, transcript variant 3 was measured. The expression of GFP alone was used as a control. The bar graph represents the mean ± SEM of four independent measurements (*n* = 4). The *p*-value was calculated by *t*-test. **(F)** The growth of yeast of BY4741 or W303 strain, with or without the deletion of the *ATE1* gene, was examined by serial dilution on agar plates for either a fermenting condition with the glucose-containing rich media (YPD), or a respiring condition with the glycerol and ethanol-based media (YPEG). **(G)** The cellular oxygen consumption rates of yeast of BY4741 or W303 strain, with or without the deletion of the *ATE1* gene. The bar graph represents the mean ± SEM of three independent measurements (*n* = 3). The *p*-value was calculated by *t*-test. **(H)** The level of membrane potential in BY4741 yeast, with or without the deletion of the *ATE1* gene, in the increasing level of CCCP, quantified using TMRM staining and flow cytometry. The bar graph represents the mean ± SD of three independent measurements (*n* = 3). The *p*-value was calculated by *t*-test.

We next examined in whole cells the steady-state levels of VDAC and TOM20, two mitochondrial markers, and did not find a significant difference between WT and *ATE1-*KO MEF (Figure 3A). This suggested that the ATE1 does not directly affect cellular mitochondrial mass. However, when we examined mitochondrial morphology by electron microscopy, we found significant differences between WT and *ATE1-*KO cells (Figure 3B). Remarkably, mitochondrial filaments in *ATE1-*KO cells were significantly shorter and wider (Figure 3C), reminiscent of the swollen mitochondria often observed in respiratory-deficient cells, as well as typical cancer cells with a glycolytic metabolic profile (Alirol and Martinou, 2006).

To assess whether any mitochondrial functional difference accompanies the observed morphological changes, we measured cellular respiration in *ATE1-*KO and WT MEF. Our data showed that *ATE1-*KO cells had a ~30% reduction in endogenous oxygen consumption rate compared to WT MEF (Figure 3D). As an essential control, we reconstituted *ATE1*-KO MEF with a recombinant *ATE1* (transcript variant 3, which was shown to have the most potent anti-tumor growth effect) at a level comparable to the endogenous protein. Recombinant *ATE1* rescued the *ATE1*-KO respiratory defects (Figure 3E). Consistent with the observations in mammalian cells, we found that the deletion of *ate1* in budding yeast also lead to slower growth in respiratory media (Figure 3F), reduced endogenous cell respiration by nearly 30%, and attenuated the mitochondrial membrane potential by more than 50% (Figures 3G,H).

### ATE1 Is Essential for the Proper Assembly, Organization, and Function of Mitochondrial Respiratory Complexes

**Figure 4:**
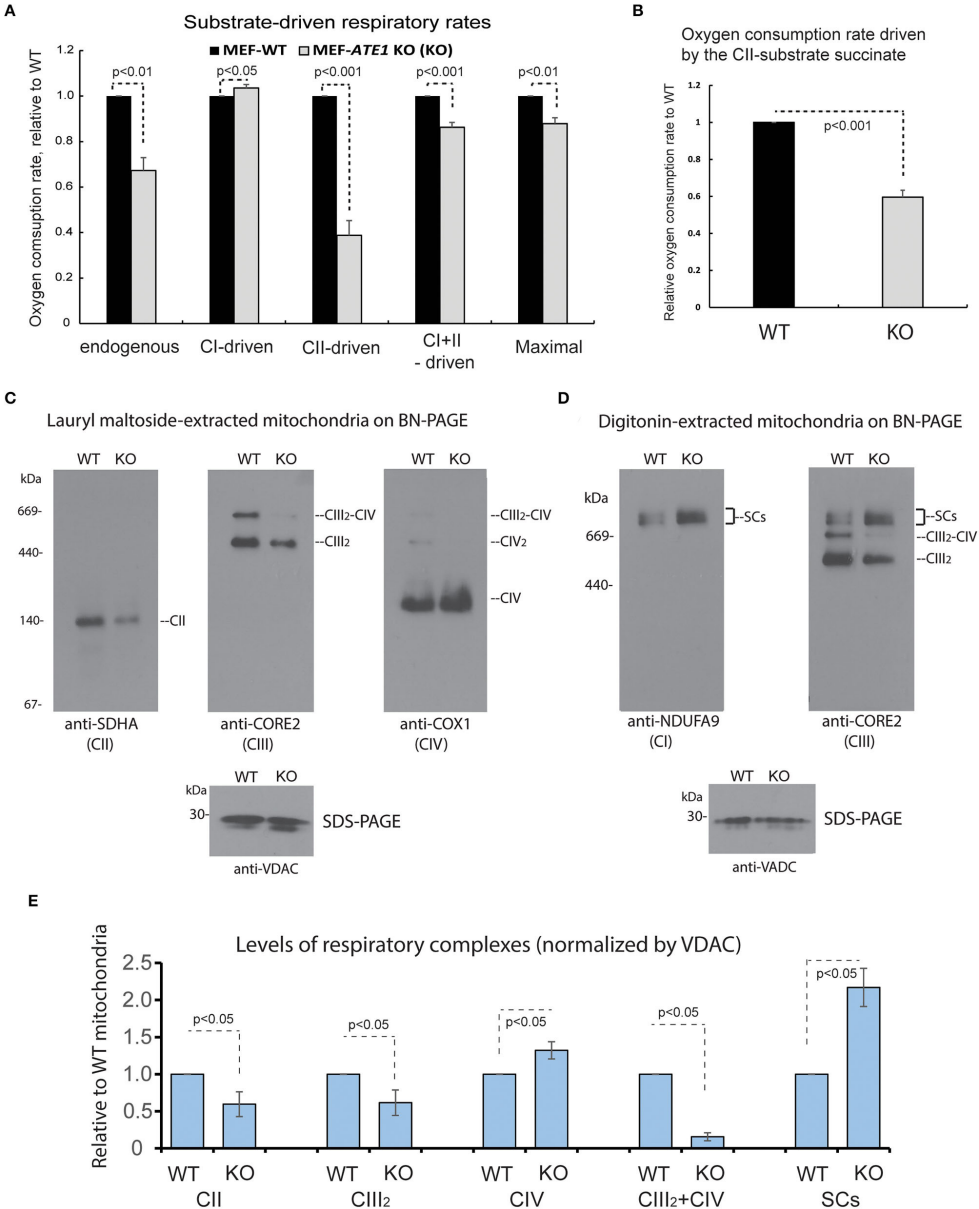
ATE1 differentially affects the composition and function of mitochondrial respiration complexes. **(A)** Oxygen consumption rates in intact whole cell (endogenous respiration) before permeabilization and CI and CII substrate-driven coupled respiration in digitonin-permeabilized WT and *ATE1-*KO (referred as KO) MEF. Glutamate plus malate were used as CI substrates, followed by succinate as a CII substrate. Maximal respiratory rates were measured upon addition of the uncoupler FCCP. The values in *ATE1-*KO cells were normalized to those in WT cells in each measurement. The bar graph represents the mean ± SEM of four independent measurements (*n* = 4). The *p*-value was calculated by *t*-test. See also Supplementary Figure 2 for representative images of the oxygen consumption curve. **(B)** Oxygen consumption rate in permeabilized cells in presence of only complex II substrate succinate in WT and *ATE1-*KO MEF. The bar graph represents the mean ± SD of four independent measurements (*n* = 4). The *p*-value was calculated by *t*-test. **(C)** Individual respiratory complexes of isolated and lauryl maltoside-extracted mitochondria were separated by blue-native polyacrylamide gel (BN-PAGE) and then examined in Western blot. SDHA, CORE2, and COX1 are established markers for complex II, III, and IV (referred as to CII, CIII, CIV in figure labeling), respectively. The CIII_2_-CIV supercomplex is also indicated. In the bottom panel, VDAC levels assessed by SDS-PAGE and immunoblotting are shown as loading control. **(D)** Similar to **(A)**, except that digitonin was used to extract mitochondrial proteins to preserve supercomplex association. NDUFA9 and CORE2 are established markers for complex I and III (CI and CIII), respectively. SCs indicates CI-containing supercomplexes (CI+CIII_2_+CIVn). **(E)** Quantitative assessment of the assembly and organization of different respiratory complexes and CI-containing supercomplexes (SCs) in mitochondria from WT and ATE1-KO MEF, extracted with lauryl maltoside or digitonin and separated by BN-PAGE. The bar graph represents the mean ± SEM of three independent repetitions (*n* = 3). The *p*-value was calculated by *t*-test.

The mitochondrial respiratory chain (MRC) is formed by four multimeric complexes (complex I to IV, CI-CIV) and two mobile electron carriers (coenzyme Q and cytochrome *c*). Electrons from the reducing equivalents NADH and FADH_2_ enter the MRC at the level of CI and CII, respectively, and are sequentially transferred until CIV, where the final electron acceptor, molecular oxygen, is reduced to water. Additionally, MRC CI, CIII, and CIV dynamically associate in supramolecular structures known as supercomplexes. To further dissect the respiratory defect observed in the *ATE1-*KO cells, we first measured oxygen consumption rates in digitonin-permeabilized cells following the addition of substrates specific for CI (glutamate-malate) or CII (succinate). When we examined the overall impact of ATE1 on mitochondrial respiration, we found that the combined rate of CI- and CII substrate-driven respiration, and the maximum respiratory capacity measured in the presence of the uncloupler FCCP, are both reduced in KO cells (Figure 4A). CI does not contribute to the deleterious effect on cellular respiration since the glutamate-malate oxidation rate is slightly but significantly higher in ATE1-KO cells than in control. In contrast, the oxygen consumption rate in the presence of succinate was lowered by nearly 50% in *ATE1*-KO cells (Figures 4A,B and Supplementary Figure 2), indicating a specific CII activity defect.

To determine the cause of the respiratory alteration, we examined the levels of individual mitochondrial respiratory complexes and supercomplexes by Blue Native-PAGE followed by immunoblotting. Consistent with the respiratory data, the steady-state levels of mitochondrial CII in *ATE1*-KO cells were found to be ~50% than in WT MEFs (Figure 4C). However, we also observed a decrease in dimeric complex III (CIII_2_) accumulation, while CIV was unaffected (Figure 4C). Moreover, an evident remodeling of the MRC organization was detected. In *ATE1*-KO cells, the levels of CI-containing supercomplexes (I+III_2_ and I+III_2_+IV_1−n_) were increased, whereas the amount of CIII that accumulated in CIII_2_ and the III_2_+IV supercomplex was attenuated (Figures 4D,E). This remodeling facilitates an increase in CI stability, presumably in an attempt to compensate for the lower levels of CII.

**Figure 5:**
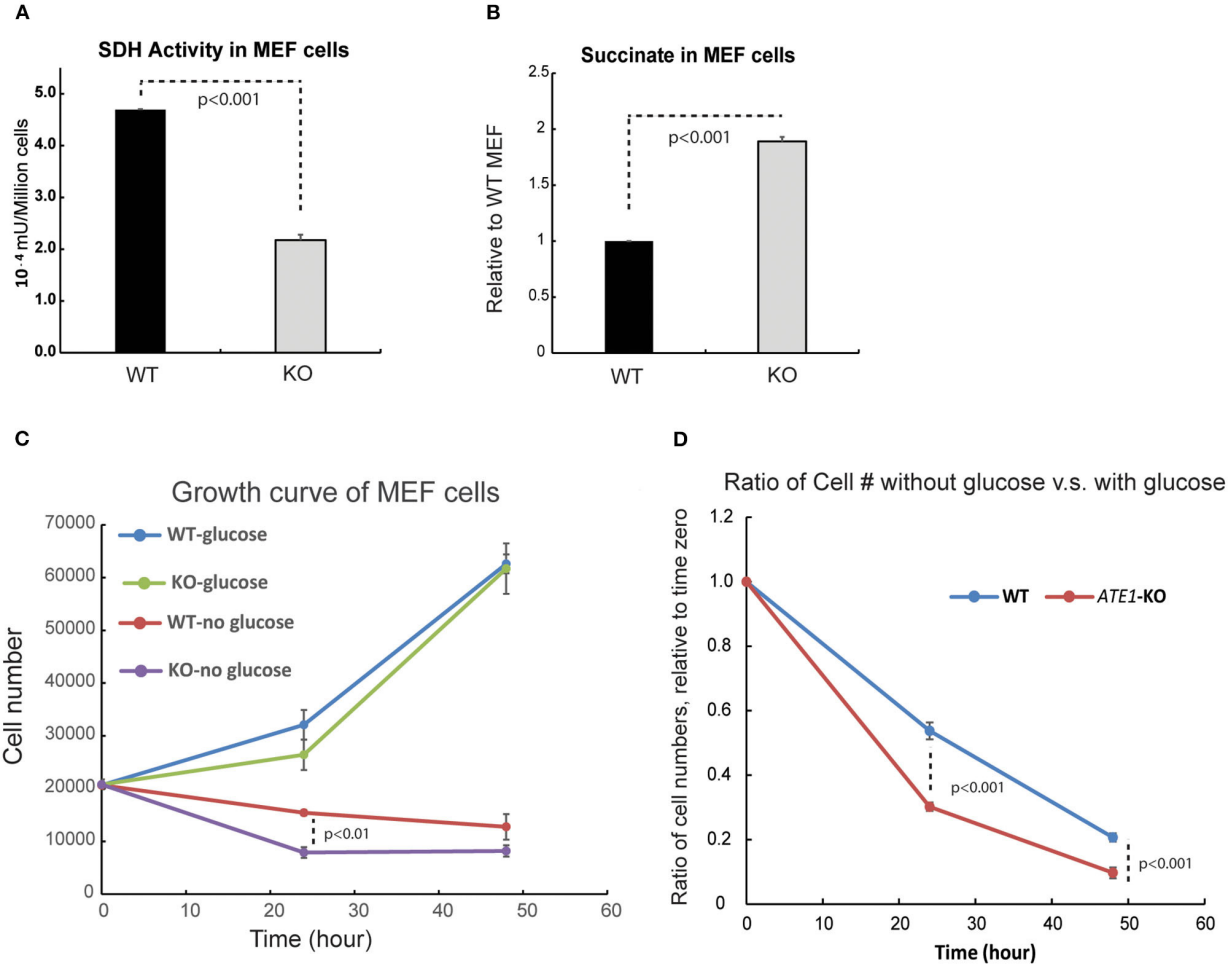
ATE1 affects the level of succinate and the cellular dependency of glucose. **(A)** The specific activity of succinate dehydrogenase (SDH) in WT and *ATE1-*KO MEF normalized by cell number. The bar graph represents the mean ± SEM of four independent measurements (*n* = 4). The *p*-value was calculated by *t*-test. **(B)** Comparison of the cellular concentrations of succinate in WT and *ATE1-*KO MEF (*n* = 3), normalized by the concentration in WT cells. The bar graph represents the mean ± SEM of three independent measurements (*n* = 3). The *p*-value was calculated by *t*-test. **(C)** The growth curves of WT and *ATE1*-KO MEF in the presence or absence of glucose. Each point represents the mean ± SEM of six independent measurements (*n* = 6). The *p*-value was calculated by *t*-test. **(D)** The sensitivities of WT and *ATE1-*KO MEF to glucose-starvation, represented by the ratio of cell number in glucose-free condition compared to that in high-glucose condition in the same time point as shown in **(C)**. Each point represents the mean ± SEM of six independent measurements (*n* = 6). The *p*-value was calculated by *t*-test.

MRC CII or succinate dehydrogenase (SDH) is also an enzyme of the TCA cycle, in which it catalyzes the conversion of succinate to fumarate. Succinate, when in excess, promotes glycolysis and therefore acts as a critical signaling component connecting the TCA cycle to glycolysis. When we measured the enzymatic activity of SDH, we found it significantly reduced in *ATE1-*KO cells (Figure 5A). Correspondingly, the level of succinate in *ATE1-*KO MEF was nearly 2-fold higher than in WT (Figure 5B). To test if this would change the balance between mitochondrial OXPHOS and glycolysis, we measured the viability of *ATE1-*KO MEF under glucose starvation and found that they are significantly more sensitive to the lack of glucose supply than WT cells (Figures 5C,D).

**Figure 6:**
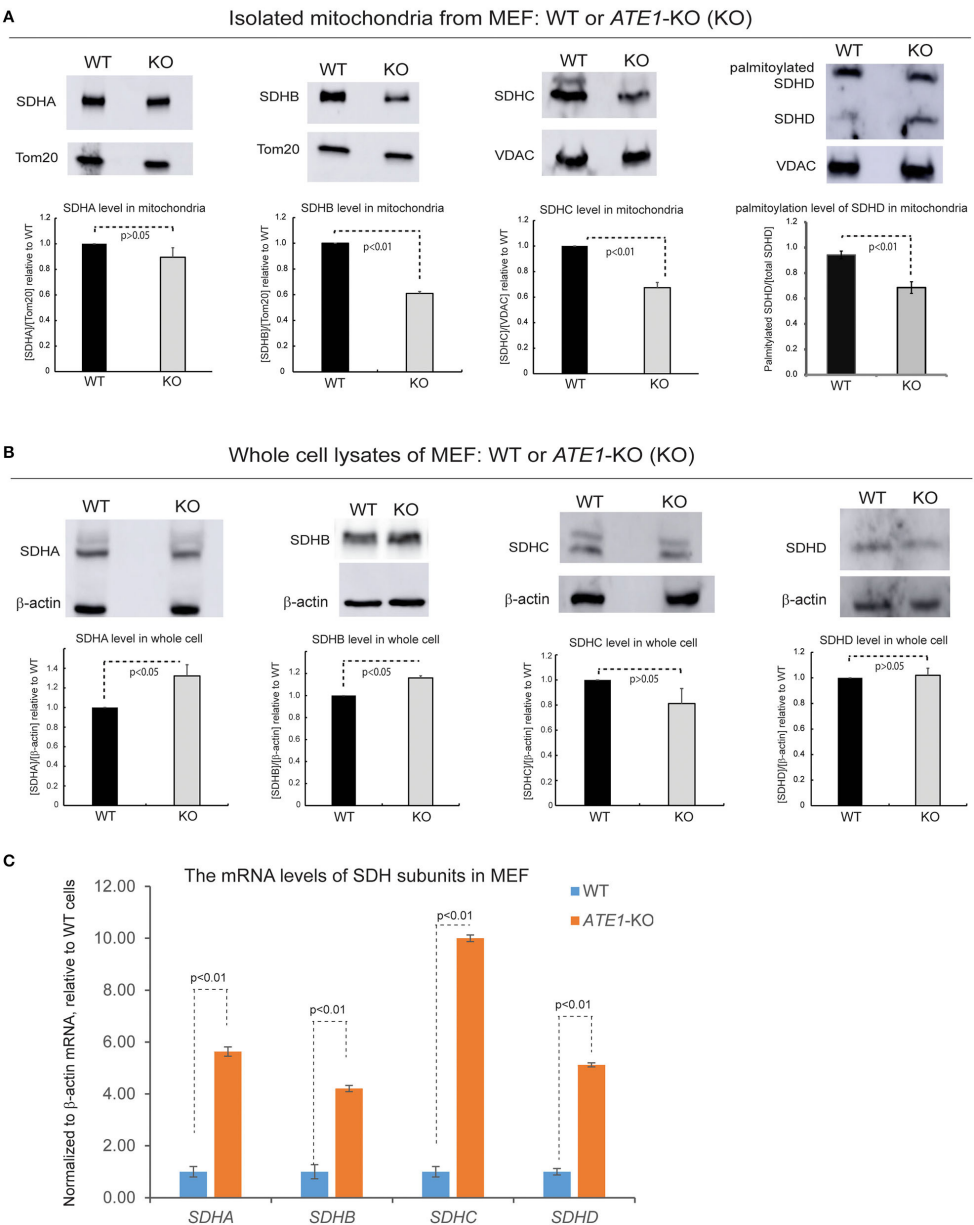
ATE1 is requited for the biogenesis of succinate dehydrogenase (mitochondrial complex II). **(A)** The protein levels of SDHA, SDHB, SDHC, SDHD in purified mitochondria from WT and *ATE1-*KO MEF. TOM20 or VDAC was used as a loading control. The bar graphs represent the mean ± SEM of three independent measurements (*n* = 3) for SDHA, SDHB, and SDHC, or five independent measurements (*n* = 5) for SDHD. The *p*-value was calculated by *t*-test. **(B)** Similar to **(A)**, except that whole cell lysates were used for analysis. Beta-actin was used as a loading control. The quantified results in the charts are shown on the lower panels. The bar graphs represent the mean ± SD of three independent measurements (*n* = 3). The *p*-value was calculate by *t*-test. **(C)** The mRNA level of *SDHA, SDHB, SDHC*, and *SDHD* in WT and *ATE1-*KO MEF measured by quantitative PCR. The β*-ACTIN (ACTB)* mRNA levels were used as loading control. In each testing group containing 4 replicates (*n* = 4), one sample from the WT cells was defined as 1.0 for the normalization of other samples. The bar graphs represent the mean ± SD. The *p*-value was calculated by *t*-test.

SDH is comprised of four subunits known as SDHA, B, C, and D, all of which are encoded in the nuclear genome and then imported into mitochondria (Van Vranken et al., 2015). When we examined the levels of these subunits in the mitochondrial fraction, we found that the levels of SDHB and SDHC, as well as the palmitoylation level of SDHD, are decreased in mitochondria in *ATE1-*KO MEF, in agreement with the lower levels of CII (Figure 6A). However, the total protein levels of these subunits in whole *ATE1-*KO cells were either unchanged or even slightly increased compared to WT cells (Figure 6B) while the transcription of SDH subunits is drastically increased in *ATE1*-KO MEF cells (Figure 6C), likely as a compensation for the functional SDH deficiency. These data indicate that ATE1 may affect the mitochondrial import and/or assembly of SDH subunits, ultimately leading to CII deficiency.

## Discussion

This study describes the discovery of uncharacterized physical and functional connections of ATE1 with the mitochondrion.

The *ATE1* gene is conserved in eukaryotes and bacteria. In eukaryotes, *ATE1* is a nuclear gene whose roles in regulating the functions and/or half-lives of cytosolic or nuclear proteins have been well-documented (Varshavsky, 2011). Here, we found several lines of evidence connecting ATE1 to mitochondria. First, phylogenetic studies pointed toward an alpha-protobacterial origin for ATE1. Second, fluorescence microscopy examinations of recombinant ATE1 expressed in MEFs and biochemical assays of endogenous ATE1 determined that a fraction of the protein resides within mitochondria. Third, the absence of ATE1 in *ATE1*-KO MEFs results in decreased MRC complex II levels and a major reorganization of the MRC into free complexes and supercomplexes. And fourth, decreased respiratory capacity and increased succinate levels in *ATE1*-KO MEFs correlate with elevated dependency on glucose.

The marked decrease in MRC CII and the concomitant rearrangement of the MRC complexes I, III, and IV into CI-containing supercomplexes, as observed in *ATE1-KO* cells, agree with the plasticity model of MRC organization. According to this model, MRC complexes exist as free entities or in SC associations in a dynamic manner to optimize electron flux from different substrates, adapting the efficiency of the respiratory chain to changes in cellular metabolism (Acin-Perez et al., 2008). Therefore, if electron-transfer from succinate-linked FADH_2_ to coenzyme Q-CIII is compromised, NADH-driven respiration would be enhanced by reorganizing the CIII and CIV complexes into I+III_2_+IV_n_ supercomplexes. In other words, we interpret the MRC complex re-organization as a consequence of the metabolic remodeling induced by the CII or SDH deficiency. However, at this stage, our studies did not disclose whether the mitochondrial fraction of ATE1 is directly responsible for the CII assembly/stability defect or whether this is also influenced by the cytoplasmic pool of the ATE1 protein that, for example, could act to regulate the import of MRC subunits into mitochondria. Nevertheless, the data in our study appear to suggest that the proposed role of ATE1 in protein degradation in the cytosol (Bachmair et al., 1986; Tasaki et al., 2012) is not very likely to play a significant role in the regulation of mitochondrial respiratory complexes. For example, we found that the transcription of all four SDH subunits is up-regulated by 4–10-folds in the *ATE1*-KO MEF. If the absence of ATE1 attenuates the degradation of SDH subunits as predicted by the N-end rule theory, one would expect a drastic increase of SDH subunit protein levels accumulating in the cytosol. However, this is not the case since the SDH subunit protein levels measured in whole cells are either unchanged or only slightly elevated. On the other hand, the increased SDH subunits transcription that we have observed in *ATE1*-KO cells is more likely a compensatory cellular response to the functional SDH deficiency.

Given that mitochondria are a major source of ROS and a central regulator of apoptosis, the role of ATE1 in this organelle may provide an explanation for its observed involvement in the stress response (Bongiovanni et al., 1999; Decca et al., 2007; Carpio et al., 2010, 2013; Lopez Sambrooks et al., 2012; Deka et al., 2016; Kumar et al., 2016; Birnbaum et al., 2019; Kim et al., 2020). Future studies will be devoted to understanding whether the translocation of ATE1 into mitochondria (or other cellular compartments such as the nucleus) is influenced by stress. Independently, our observations have profound implications in physiological processes such as embryo/tissue development and aging, and in disease conditions such as cancer and inflammation, in which the involvement of ATE1 is poorly understood.

*ATE1* is downregulated in multiple types of cancer, including those observed in the kidney, prostate, and colorectum (Rai et al., 2015; Birnbaum et al., 2019). Therefore, our findings offer a novel explanation for the commonly seen mitochondrial dysregulation in these cancers (Zhong et al., 2005; Rai et al., 2015). Notably, in most normal cells, the balance between glycolysis and mitochondrial respiration is essentially maintained by reciprocal feedback pathways, including the allosteric inhibitory effect of mitochondrion-generated ATP on AMP-activated protein kinase or citrate on phosphofructose kinase (Gogvadze et al., 2008). However, the downregulation of mitochondria function in cancer is not necessarily a consequence of the increase in glycolysis known as the Warburg effect (Alirol and Martinou, 2006; Viale et al., 2015). It has been proposed that the existence of mutations in genes coding for mitochondrial proteins and/or metabolic enzymes may contribute to tumorigenesis. These include the genes encoding SDH subunits, which are often considered as tumor suppressors (Buffet et al., 2020). SDH mutations result in the accumulation of succinate and subsequent stabilization of HIF-1α (Majmundar et al., 2010), an essential regulator of glycolysis and stress response, and relevant to tumor growth (Tran et al., 2016). According to our results, the downregulation of ATE1 in multiple cancers may provide another pathway to affect the function of SDH without involving mutations on SDH genes, which may be an important regulatory mechanism in carcinogenesis. The potential effects of ATE1 on HIF1α and glycolysis also warrant future investigations.

The role of ATE1 in metabolic regulation may be also relevant in injury response and inflammation since increased arginylation is often observed in these scenarios (Shyne-Athwal et al., 1988; Luo et al., 1990; Jack et al., 1992; Xu et al., 1993; Wang and Ingoglia, 1997). Cells of the innate immune system recognize pathogen-associated molecular patterns via receptors such as Toll-like receptors (TLRs), present on their cell surface and in the cytosol (Janeway and Medzhitov, 2002). In resting conditions, these cells are relatively inactive; however, they respond rapidly upon recognition of foreign material, by adapting to the significant metabolic demands (Pearce and Pearce, 2013). For example, stimulation of dendritic cells and macrophages often result in decreased OXPHOS, which is normally used under resting conditions, and an increase in glycolysis and the pentose-phosphate pathway (Tannahill et al., 2013). There are interesting parallels between tumors and, for example, lipopolysaccharide-activated macrophages concerning succinate metabolism. Succinate levels also increase in macrophages, where a similar process of HIF-1α stabilization by succinate occurs (Mills and O'Neill, 2014). Whether ATE1 could be involved in these processes deserves further investigations.

In summary, our findings open a new paradigm for studies of metabolism and associated diseases. ATE1 may play an instrumental role in contributing to the metabolic shift from OXPHOS to glycolysis seen in cancer and inflammation, and the adaptation to oxidative, hypoxic, or thermal stresses. Future efforts will be devoted to understanding whether ATE1 translocates to mitochondria upon stress and identify the ATE1 substrates within mitochondria to disclose the precise role/s ATE1 performs in the organelle.

## Materials and Methods

### Mammalian Cells and Media

Immortalized mouse embryonic fibroblasts (MEFs) that are either wild-type or genomic knockout for *ATE1* (*ATE1-*KO) were a gift from Dr. Anna Kashina (University of Pennsylvania), prepared as described elsewhere (Zhang et al., 2012). The human embryonic kidney cell line (HEK 293T; clone T7) was obtained from ATCC.

For routine growth and maintenance, unless otherwise indicated, mammalian cells were cultured in media containing high-glucose DMEM supplemented with 1 mM pyruvate and glutamine (Gibco, Cat# 10569) with 10% FBS (HyClone, Cat# SH30910.03). No antibiotics were used for the cell culture to minimize interference with cellular metabolism, and the cultured cells were periodically checked for bacterial or mycoplasma contamination. The cells were cultured in a 5% CO_2_ incubator at 37°C, unless otherwise indicated. To minimize any effects of contact inhibition, only actively growing cells in culture density of <50% confluency were used for any test in this study, unless otherwise indicated.

### Yeast Strains

The *Saccharomyces cerevisiae* strains used in this study include BY4741 (*MATa his3*Δ*1 leu2*Δ*0 met15*Δ*0 ura3*Δ*0*) and W303-1A (*MATa leu2-3,112 trp1-1 can1-100 ura3-1 ade2-1 his3-11,15 ybp1-1*). They are both obtained from Open Biosystems. A strain carrying a null *ate1*Δ:KanMX cassette in the BY4741 background was obtained from Open Biosystems. To create *ate1-*deletion in the W303-1A yeast, we applied the knockout cassette amplified from the *ate1*Δ:KanMX in BY4741-strain yeast with two primers as described before (Kumar et al., 2016):

ATE1300UP: ATGGTGCTGTGCTTGTAATTGCC

ATE1300DOWN: GCTCATCAAAAACTAAGAATAAGAG

The strain with a GFP fused to the C-terminal end of endogenous Ate1 in the native chromosome locus in BY4741 genetic background was obtained from Open Biosystems. The identity of each knockout strain listed above was confirmed with PCR genotyping.

### Culture of Yeast

Yeast culture media were prepared as described below:

YPD: 2% glucose, 1% yeast extract, 2% peptone,

SD (Synthetic Defined) Medium (per 1,000 ml): Yeast Nitrogen Base, 1.7 g, Ammonium sulfate, 5 g, Dextrose/galactose/raffinose, 20 g, required amino acids, 50 mg, uracil (if required), 50 mg.

YPEG: 1% yeast extract, 2% peptone, 3% (v/v) glycerol, and 2% ethanol.

For solid media plates, 2% agar was added to the liquid media.

Strains grown in liquid and solid media were incubated at 30°C unless otherwise indicated.

For most serial dilution growth assays, a single colony of yeast was inoculated in liquid medium and allowed to grow to the log phase before spot platting of serial dilutions as described elsewhere (Kumar et al., 2012).

### Antibodies

Primary antibodies used in this study are (unless otherwise indicated): rat anti ATE1 (EMD-Millipore, Billerica, MA, Cat# MABS436, clone 6F11), mouse anti-GFP (Roche Diagnostics, Indianapolis, IN, Cat# 11814460001), mouse anti porin/VDAC1 (Abcam, Cambridge, MA, Cat# ab14734), anti TOM20 (Santa Cruz Biotechnology, Dallas, TX, Cat# SC11415), anti-beta-actin (Sigma-Aldrich, St. Louis, MO, Cat# A1978), mouse anti-beta-tubulin (Sigma-Aldrich, St. Louis, MO, Cat# T5201), anti-histone H3 (Abcam, Cambridge, MA, Cat# ab1971), rabbit anti-SDHA (One World Lab, San Diego, Ca, Cat# 53800), mouse anti-SDHB (Abcam, Cambridge, MA, Cat# ab14714), rabbit anti-SDHC (Abcam, Cambridge, MA, Cat# ab155999), rabbit anti-SDHD (EMD-Millipore, Billerica, MA, Cat# ABC835), rabbit anti-Hexokinase2 (One World Lab, San Diego, Ca, Cat#55911), mouse anti-PHD2 (Santa Cruz Biotechnology, Dallas, TX, Cat# SC271835).

Secondary antibodies used in this study are (unless otherwise indicated): Anti-mouse-HRP (Pierce, now thermos Scientific, Cat # 31430), Anti-rabbit-HRP (Thermo Fisher Cat # 65-6120), Anti-Rat-HRP (BioLegand, Cat # 405405), Anti-mouse-Alexa 488 (Molecular Probe /Invitrogen, Cat # A21202), Anti-rabbit-Alexa 488 (Molecular Probes/ Invitrogen, Cat # 21206), Anti-Rat-Alexa 594 (Molecular Probe/Invitrogen, Cat # A21209).

### SDS-PAGE and Western Blot

Cell lysate or protein samples were generally prepared in SDS-loading buffer and boiled for 10 min for denaturing. For analysis of membrane-associated proteins, samples were first dissolved in 8M urea/PBS and then added SDS-loading buffer and denatured at 55°C for 10 min. The proteins were separated by electrophoresis in 4–20 or 10% SDS-PAGE (unless otherwise indicated) as needed. The proteins were then transferred to nitrocellulose or PVDF membranes for Western Blot analysis. The protein bands were examined with Chemifluorescence visualization utilizing the HRP conjugated on secondary antibodies and reagents provided in the BM Chemifluorescence Western Blotting Kit Mouse/Rabbit (Roche) or the SuperSignal West Femto Chemiluminescence Kit (Pierce). The chemiluminescent signals were either documented on film (Denville) or by GE Amersham Imager model 600. The films were scanned by an Epson Perfection 2400 photo/film scanner with at least 1,200 dpi resolution to convert into digital forms and then analyzed with Image J (NIH). For images documented by the GE imager, an ImageQuant TL software pack (v8.1) and its 1D gel analysis module were used to examine the intensity of signals by densitometry.

### Analysis of Mitochondrial Complex on Blue Native PAGE (BN-PAGE)

Cells were permeabilized in PBS with 2 mg/ml digitonin (Sigma-Aldrich, Cat# D141) at 4°C for 10 min. Cells were centrifuged at 10,000 × g for 5 min at 4°C and washed twice with PBS. Cell pellets were resuspended in 1.5 M aminocaproic acid, 50 mM Bis–Tris pH 7.0, and total protein concentration was determined by the Folin method (Lowry et al., 1951). Proteins were extracted either with 1% lauryl maltoside (for monomeric complex analysis) or 1% digitonin (for supercomplex analysis). After centrifugation at 22,000 × g for 30 min at 4°C, a buffer containing 750 mM aminocaproic acid, 50 mM Bis-Tris pH 7.0, 0.5 mM, EDTA, and 5% Serva blue G was added to the clarified extracts. Samples were loaded on a linear 3–12% polyacrylamide blue native gel (Invitrogen), transferred to PVDF membrane, and analyzed by immunoblotting with the following antibodies: anti-COX1 (Abcam, Cat#Ab14705), anti-COX3 (Abcam, Cat# Ab110259), anti-NDUFA9 (Abcam, Cat# Ab14713), anti-CORE2 (Abcam, Cat# Ab 14745), anti-SDHA (Abcam, Cat# Ab14715), anti-porin/VDAC (Abcam, Cat# Ab14734), anti-TOM20 (Santa Cruz Biotechnology, Cat# sc-11415), second antibodies anti-mouse HRP (Rockland, Cat# 610-4302), anti-rabbit HRP (Rockland, Cat# 611-1302).

### Preparation of Cells Stably Expressing Recombinant ATE1 Proteins

*ATE1* KO MEF stably transfected with ATE1-1-GFP, ATE1-2-GFP, ATE1-3-GFP, and ATE1-4-GFP was generated as described previously (Zhang et al., 2015) except cells were enriched with FACS without puromycin selection. Briefly, retroviruses carrying different C-terminal GFP tagged isoform of Ate-1 were constructed by transfecting HEK293T cells with the low-expression pBabe-Puro vectors carrying coding sequence for the desired isoform and the vectors of GAG-Pol and VSV-G vector. The viruses were then allowed to infect *ATE1* KO MEF in the presence of 10 mg/mL polybrene. Successfully transfected MEF were enriched by fluorescence sorting.

### Cell Counting and Cell Size Measurement

The numbers of resuspended cells in solution were counted with an automated Biorad TC20 cell counter, and dead cells were excluded with trypan blue unless otherwise indicated. The diameters of the resuspended cells were also measured by the built-in feature of the cell counter. All peaks of size distributions and the associated cell numbers displayed by the counters were used to calculate the average diameter of the cell.

### Measurements of Whole-Cell and Mitochondrial Respiration Activity in Mammalian Cells

For the tests described below, we used actively growing cell cultures at a confluency lower than 50%. The measurements of whole-cell and mitochondrial respiration rates were done according to a protocol published elsewhere (Barrientos et al., 2009). In brief, cells were collected by trypsinization, neutralized in FBS-containing medium, and washed with DPBS. To measure whole-cell respiration, the cells were resuspended in RPMI 1640 medium with L-Glutamine and 25 mM HEPES (Sigma-Aldrich, Cat# SLM-140) at 37°C. The numbers of live cells were counted using Trypan blue on a TC-20 automated cell counter (Bio-Rad). The concentration of all cell lines used in the same test were adjusted to 4 × 10^6^ cells/ml and then transferred to the chamber of a polarographic apparatus with a Clark-type O2 electrode (Hansatech, Oxygraph plus system) for measurement of cell respiration at 37°C. At the end of the measurement, KCN (Sigma-Aldrich, Cat# 60178) was added to the cell suspension to a final concentration of 2 mM to inhibit cell respiration. KCN-sensitive cellular respiratory rates were obtained by subtracting oxygen consumption rates before and after KCN addition.

To measure substrate-driven mitochondrial respiration, the cells were resuspended in warm (37°C) permeablized-cell respiration buffer (PRB) containing 0.3 M mannitol, 10 mM KCl, 5 mM MgCl_2_, 0.5 mM EDTA, 0.5 mM EGTA, 1 mg/ml BSA and 10 mM KH_3_PO4 (pH 7.4). The numbers of live cells were counted using Trypan blue on a TC-20 automated cell counter (Bio-Rad). The concentrations of each cell type in the same test were adjusted to 4 × 10^6^ cells/ml. An aliquot of the cell suspension was supplemented with freshly prepared hexokinase (Sigma-Aldrich, Cat# H-5500) to 10 U/mL, and ADP to 2 mM and 1 mL of the cell suspension was transferred to the polarographic chamber/oxygraph containing a Clark-type O_2_ electrode (Hansatech, Oxygraph plus system) with a temperature setting of 37°C. The whole-cell respiration was measured before digitonin was added.

To measure substrate-driven respiration, the cell membrane was permeabilized by adding freshly prepared digitonin in optimized ratios of 20 μg per 10^6^ cells for WT MEF, or 10 μg per 10^6^ cells for *ATE1-*KO MEF. The substrate-driven oxygen consumption rate was then examined by adding specific MRC substrates using a Hamilton microsyringe. To measure complex I-driven respiration, we added 5 mM each of glutamate (Sigma-Aldrich, Cat# G8415) and malate (Sigma-Aldrich, Cat# M1124). To measure complex II, we used 10 mM succinate (ICN Biomedicals, Cat# 102972). To measure uncoupled respiration, we added 3 μM carbonilcyanide p-triflouromethoxyphenylhydrazone (FCCP) (Sigma-Aldrich, Cat# C2920), and to assess the specificity of the measurements, we inhibited respiration with 2 mM of the CIV inhibitor potassium cyanide (KCN) (Sigma-Aldrich, Cat# 60178).

### Isolation of High-Purity Mitochondria From Cultured Cells for Protein Analysis

Mitochondria were purified from exponentially growing cultured cells based on the Gaines method, updated by Enriquez's group (Fernandez-Vizarra et al., 2010). In brief, cells were harvested by trypsinization and washed twice with cold DPBS before being resuspended in swelling buffer (10 mM Tris–HCl, pH 7.4, 10 mM KCl, 0.5 mM MgCl_2_) and incubation on ice for 5 min. The cells were then homogenized with Teflon-glass douncer until at least 75% of the cells were broken. The homogenized lysate was then mixed with sucrose solution to reach a final concentration of 0.25 M of sucrose and then centrifuged at 600 × g for 5 min at 4°C twice to remove cell debris. The resulting supernatant was centrifuged at 7,000 × g for 10 min at 4°C to pellet the mitochondrial fraction. The pellet was washed with the STE buffer (0.32 M sucrose, 1 mM EDTA, and 10 mM Tris–HCl, pH 7.4) and centrifuged again at 7,000 × g for 10 min at 4°C. The pellet was gently resuspended in freshly prepared, ice-cold mitochondria resuspending buffer (MRB) (250 mM mannitol, 5-mM HEPES (pH 7.4), and 0.5-mM EGTA. The mixture was layered on top of Percoll medium (225-mM mannitol, 25-mM HEPES (pH 7.4), 1-mM EGTA, and 30% Percoll (vol/vol). Additional MRB buffer was added to fill the volume of the centrifuge tube before being centrifuged in a swing-bucket rotor at 95,000 × g for 30 min at 4°C. A Beckman Coulter Optima L-100 XP ultracentrifuge with an SW40 rotor (Beckman, Fullerton, CA, USA) was used in this study. The purified mitochondria, which was present in a semi-transparent layer directly above the pellet on the bottom, was then recovered.

### Measurement of Endogenous Cell Respiration in Yeast Cells

The cell respiration of yeasts was assayed polarographically using a Clark-type oxygen electrode (Hansatech Instruments, Norfolk, UK) at 30°C as described elsewhere (Barrientos et al., 2002). The specific activities reported were corrected for KCN-insensitive respiration.

### Measurement of Membrane Potential in Yeast Cells

The measurement of mitochondrial membrane potential (Ψ_m_) in yeast was performed as described elsewhere (Ocampo et al., 2012). In brief, yeast cells were incubated with 5 μM TMRM of Tetramethyl Rhodamine Methyl Ester (TMRM), a cell-permeant, cationic, fluorescent dye that is readily sequestered by viable mitochondria, at 30°C for 30 min, as reported (Nicholls and Ward, 2000). The cells were then washed twice in PBS and were analyzed with flow cytometry analysis on a Becton Dickinson (BD) FACSAria^TM^ II Flow Cytometer. Excitation was performed at 532 nm; emission was detected using a 25 nm bandpass filter centered at 575 nm (Becton Dickinson, NJ, USA). Dissipation of Ψ_m_ causes TMRM to leak out of mitochondria into the cytosol, where TMRM became unquenched, producing an increase in fluorescence (Nicholls and Ward, 2000). To confirm the mitochondrial specificity of the signal of TMRM, ionophore carbonyl cyanide m-chlorophenyl hydrazone (CCCP), which dissipates the membrane potential, was applied to treat the cells as controls.

### Measurement of Cellular Succinate Levels

Actively growing cells below 50% confluency were used. Cells were harvested by trypsin and then immediately washed three times with ice-cold DPBS. The numbers, viability, and diameters of the resuspended cells were measured on the Biorad TC20 automated cell counter. The same number of cells (40 million cells in this test) were used for each cell type. The cells were collected by centrifugation at 2,500 × g at 4°C for 3 min. The pellets were weighted and 20 volumes of ice-cold H_2_O was added. The cells were then lysed by brief sonication, and the cell debris was removed by centrifugation at 20,000 × g at 4°C for 10 min. The supernatant was further centrifuged at 100,000 × g at 4°C for 1 h to remove large cellular complexes. The subsequent supernatant was then filtered through a 10K MWCO membrane (Thermo Scientific, Rockford, IL, Cat#PI88513) by centrifugation at 4°C to remove large proteins. The filtrate protein concentration was quantified by the Bradford assay using reagents from Bio-Rad (Cat# 500 0205). The cell lysate filtrate was then extracted by acetonitrile. The resulting supernatant was dried under N_2_ and then derivatized by HCl n-butanol. Upon being dried under N_2_, the derivative precipitation was reconstituted with 80% methanol and loaded in LC-MS/MS. The level of succinic acid in the filtrate was quantified by Agilent triple quant LC-MS/MS. The final cellular concentration of succinic acid was normalized by cell number, cell volumes, dilution factor, and the protein concentration in the filtered lysate.

### Mitochondrial Proteinase K Protection Assay

The experiment was performed according to the previously published method (Clemente et al., 2013). In brief, purified mitochondria were resuspended in a buffer containing 10 mM Tris-HCl, pH 7, 10 mM KCl, 0.15 mM MgSO_4_, and 0.25 M sucrose. As control, one sample was subjected to brief sonication to break the membranes. All samples were then incubated in ice for 45 min in the presence of proteinase K at a final concentration of 0, 0.32, or 0.64 μg/ml. Mitochondria were recovered by centrifugation at 8,000 × g for 15 min at 4°C and analyzed by Western blotting.

### Glucose Starvation Assay

MEF cells (WT or *ATE1-*KO) were cultured in high-glucose DMEM containing 25 mM glucose and 1 mM pyruvate (Gibco Cat#10569) supplemented with 5% FBS (Hyclone, Cat# SH30910.03) for several generations. Immediately before the experiment, the cells were split and cultured for one generation (24 h) in the same high-glucose DMEM except with 5% dialyzed FBS (Life Technologies Cat# 26400-044). At the time of the experiment, the cells were trypsinized, washed with DPBS, and then resuspended in either starving media with glucose-free, pyruvate-free DMEM (Gibco, Cat# 11966) and 5% dialyzed FBS, or non-starving media with high-glucose, 1 mM pyruvate DMEM (Gibco Cat#10569) and 5% dialyzed FBS. The cells were then inoculated into 6-cm culture dishes with 50,000 cells per dish so that the cells would stay at a non-confluent culture density through the duration of tests. Live cells that remained attached to the plate were then counted at given time points, using trypan blue to exclude dead cells.

### Microscopy

Optical and fluorescent images of cells were captured on a Zeiss Observer equipped with a series of objectives and Zen Pro software. Analysis of the images were performed with the Zen Pro software.

### Image Processing

The images were processed in Adobe Photoshop by adjusting the display levels while preserving the linearity of the signals. The figures were assembled in Adobe Illustrator.

### Bioinformatic Analysis

The phylogenetic tree of the ATE1 protein was calculated based on amino acid sequences with Clustal Omega program on the Uniprot website. The detailed alignment of amino acid sequences in ATE-C domain (Pfam ID: PF04377) between two species was performed with NCBI BLASTp. The sunburst graph showing the distribution of ATE-C domain (Pfam ID: PF04377) was generated with corresponding tools from pfam.xfam.org hosted by EMBL-EBI.

### RNA Isolation and Quantitative PCR

RNA was extracted using Quick-RNA MiniPrep Kit (Genesee Scientific, Cat #: 11-328). The corresponding cDNA was prepared by using Superscript First-strand RT-PCR kit (Invitrogen, Cat#: 11904-018). Quantitative real-time PCR was performed by using SsoAdvanced™ Universal SYBR® Green Supermix (Biorad, Cat#: 1725271) on a CFX Connect Real-Time PCR machine (Biorad). The samples were run in multiple replicates on Hard- Shell PCR 96 well plates from Biorad (Cat#: HSP9601). PCR conditions: initial denaturation for 30 s at 95°C, followed by 40 cycles with 95°C for 15 s and 58°C for 1 min. After each run, a melting curve was measured to confirm the specificity of the amplification. The relative expression of the SDH subunits were calculated by delta Ct method. The mRNA of β-actin (ACTB) was used as housekeeping gene/reference gene for loading controls.

The primers targeting different subunits of Succinate dehydrogenase and β-actin are listed below.

**SDHA_qPCR_F—GCTCCTGCCTCTGTGGTTGA**
**SDHA_qPCR_R—AGCAACACCGATGAGCCTG**
**SDHB_qPCR_F—TGCGGACCTATGGTGTTGGATG**
**SDHB_qPCR_R—CCAGAGTATTGCCTCCGTTGATG**
**SDHC_qPCR_F—TGCTCCTTTGGGAACCACAGCT**
**SDHC_qPCR_R—GCAAACGGACAGTGCCATAGGA**
**SDHD_qPCR_F—GGTTGTCAGTGTTCTGCTCTTGG**
**SDHD_qPCR_R—GTCGGTAACCACTTGTCCAAGG**
**β-actin_qPCR_F—CAGCTGAGAGGGAAATCGTG**
**β-actin_qPCR_R—CGTTGCCAATAGTGATGACC**

### Statistical Analysis

The statistical significance for comparison of quantitative assessments performed in WT and *ATE1*-KO MEF or yeast cells was estimated by the *Student t*-test. The data distribution was considered one-side or two-side based on the presence of the error bars in the displayed data for comparison. Error bars represent standard error of the mean (SEM) or standard deviation (SD), as indicated in the figure legends. A minimum *p*-value of 0.05 was considered significant.

## Data Availability Statement

The original contributions presented in the study are included in the article/Supplementary Materials, further inquiries can be directed to the corresponding author/s.

## Author Contributions

FZ conceptualized, initiated, and supervised the project. FF designed and supervised most of the mitochondria-related experiments and performed some of them. CJ performed most of the experiments. BM, DP, AK, and WM participated in the performance of some of the experiments under the supervision of FZ and/or FF. BA performed the succinate measurement supervised by JH. DI participated in the bioinformatic analysis. AB and TL participated in the design of metabolism-related experiments. CJ and FZ wrote the manuscript. FF, AB, and DI edited the manuscript. All authors read and approved the manuscript.

## Conflict of Interest

The authors declare that the research was conducted in the absence of any commercial or financial relationships that could be construed as a potential conflict of interest.

## Abbreviations

ATE1: arginyltransferase 1
GFP: green-fluorescence protein
HFF: human foreskin fibroblasts
KD: knockdown
KO: knock-out
MEF: mouse embryonic fibroblasts
WT: wild-type.
