# Supplementary material for: Regulation of Mitochondrial Respiratory Chain Complex Levels, Organization, and Function by Arginyltransferase 1
Source: Front Cell Dev Biol. 2020 Dec 21;8:603688. doi: 10.3389/fcell.2020.603688 (PMC7779560; doi:10.3389/fcell.2020.603688)
Supplement: Supplementary file 1 [file Presentation_1.pdf]

Supplementary File to "Regulation of mitochondrial respiratory chain complex levels, organization and function by arginyltransferase 1"

Supplemental Figure S1

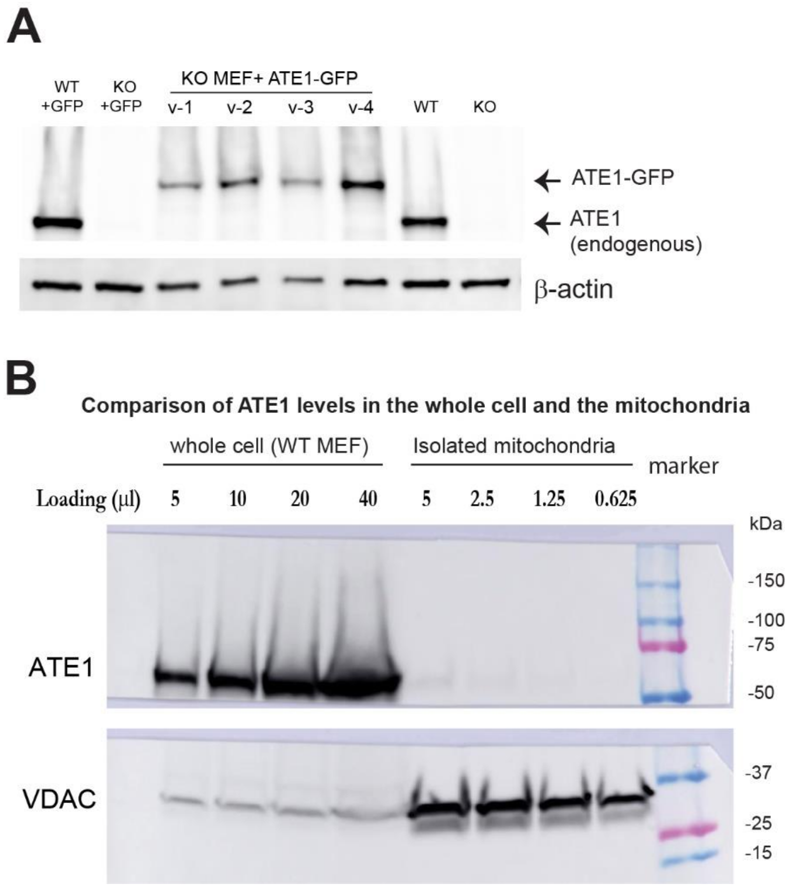

Supplemental Figure S1, related to Figure 1.

A) Immunoblot assessment of the steady-state levels of ATE1 in WT or *ATE1*-KO (KO) MEF cells that are either untreated, or stably transduced with different GFP-fused mouse ATE1 splice variants (v-1 to v-4) or GFP alone with retroviral vector. The ATE1 antibody (from Millipore, clone # 6F11) recognizes all four known mouse ATE1 splice variants (v1-v4). Note the size difference between the endogenous ATE1 and the GFP-fused recombinant ATE1. An antibody against beta-actin was used as a loading control.

B) Comparison of the ATE1 level in whole cells and isolated mitochondria in WT MEF. by different volumes of lysate, in which the cell pellets were resuspended in loading buffer by a 10x weight to volume dilution. The ratio of ATE1 *v.s.* VDAC was used to calculate the difference between the whole cell and the mitochondria. Based on these data, it is estimated that about 0.5% of total ATE1 is associated with mitochondria.

Supplemental Figure S2

Representative oxygen consumption curves of WT and *ATE1*-KO MEF measured in the polarography

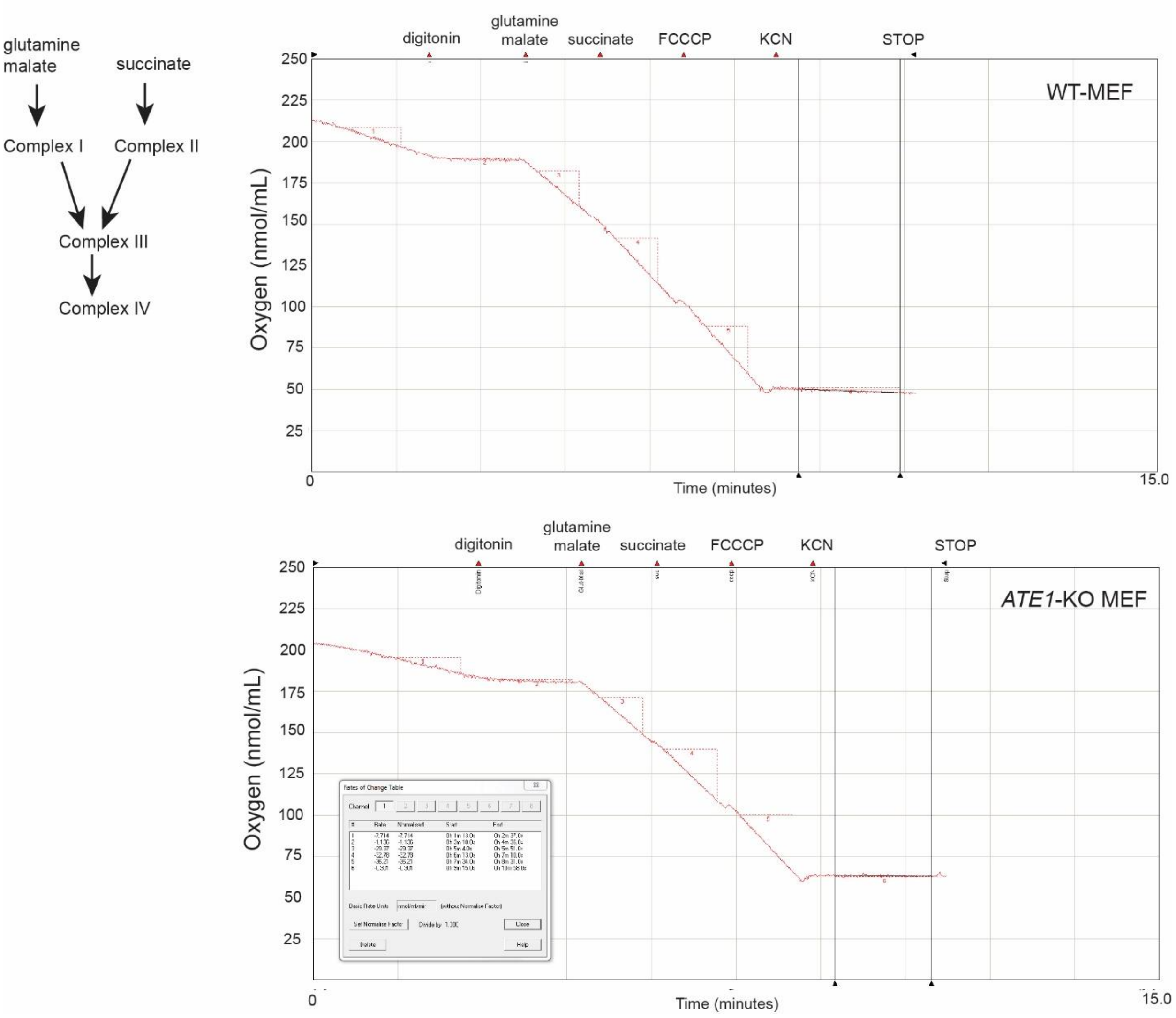

Supplemental Figure S2. Related to Figure 2.

Representative oxygen consumption trace of WT and *ATE1*-KO MEF measured by polarography, with the sequential addition of the detergent digitonin, the complex I substrates glutamate and malate, the complex II substrate succinate, the mitochondrial uncoupler FCCP, and the CIV inhibitor KCN.
